# Hypoxia-inducible factor activation promotes osteogenic transition of valve interstitial cells and accelerates aortic valve calcification in a mice model of chronic kidney disease

**DOI:** 10.3389/fcvm.2023.1168339

**Published:** 2023-06-02

**Authors:** Dávid Máté Csiki, Haneen Ababneh, Andrea Tóth, Gréta Lente, Árpád Szöőr, Anna Tóth, Csaba Fillér, Tamás Juhász, Béla Nagy, Enikő Balogh, Viktória Jeney

**Affiliations:** ^1^ MTA-DE Lendület Vascular Pathophysiology Research Group, Research Centre for Molecular Medicine, Faculty of Medicine, University of Debrecen, Debrecen, Hungary; ^2^ Doctoral School of Molecular Cell and Immune Biology, Faculty of Medicine, University of Debrecen, Debrecen, Hungary; ^3^ Department of Biophysics and Cell Biology, Faculty of Medicine, University of Debrecen, Debrecen, Hungary; ^4^ Department of Anatomy, Faculty of Medicine, University of Debrecen, Debrecen, Hungary; ^5^ Department of Laboratory Medicine, Faculty of Medicine, University of Debrecen, Debrecen, Hungary

**Keywords:** hypoxia, valve interstitial cell, osteogenic differentiation, valve calcification, hypoxia inducible factor, chronic kidney disease, reactive oxygen species

## Abstract

**Introduction:**

Valve calcification (VC) is a widespread complication in chronic kidney disease (CKD) patients. VC is an active process with the involvement of *in situ* osteogenic transition of valve interstitial cells (VICs). VC is accompanied by the activation of hypoxia inducible factor (HIF) pathway, but the role of HIF activation in the calcification process remains undiscovered.

**Methods and result:**

Using *in vitro* and *in vivo* approaches we addressed the role of HIF activation in osteogenic transition of VICs and CKD-associated VC. Elevation of osteogenic (Runx2, Sox9) and HIF activation markers (HIF-1*α* and HIF-2*α*) and VC occurred in adenine-induced CKD mice. High phosphate (Pi) induced upregulation of osteogenic (Runx2, alkaline-phosphatase, Sox9, osteocalcin) and hypoxia markers (HIF-1*α*, HIF-2*α*, Glut-1), and calcification in VICs. Down-regulation of HIF-1*α* and HIF-2*α* inhibited, whereas further activation of HIF pathway by hypoxic exposure (1% O_2_) or hypoxia mimetics [desferrioxamine, CoCl_2_, Daprodustat (DPD)] promoted Pi-induced calcification of VICs. Pi augmented the formation of reactive oxygen species (ROS) and decreased viability of VICs, whose effects were further exacerbated by hypoxia. N-acetyl cysteine inhibited Pi-induced ROS production, cell death and calcification under both normoxic and hypoxic conditions. DPD treatment corrected anemia but promoted aortic VC in the CKD mice model.

**Discussion:**

HIF activation plays a fundamental role in Pi-induced osteogenic transition of VICs and CKD-induced VC. The cellular mechanism involves stabilization of HIF-1*α* and HIF-2*α*, increased ROS production and cell death. Targeting the HIF pathways may thus be investigated as a therapeutic approach to attenuate aortic VC.

## 1. Introduction

Vascular calcification and valvular heart disease are highly prevalent in patients with chronic kidney disease (CKD). In particular, the prevalence of valve calcification (VC) is eight times higher in end stage renal disease patients undergoing hemodialysis than in the general population (1). Aortic and mitral valves are affected most frequently, and calcification of both valves arises 10–20 years sooner in CKD patients compared with subjects with normal kidney function (1–3). Hyperphosphatemia is a critical etiopathogenic factor in CKD-associated vascular and valvular calcification (4–6).

Heart valves are avascular, though metabolically active tissues, composed of an outer monolayer of valve endothelial cells and several internal layers of valve interstitial cells (VICs) (7). For a long time VC was considered as a passive deposition of calcium-phosphate which supposition was challenged by studies showing the existence of osteoblast-like and osteoclast-like cells in human aortic valve leaflets (8, 9). About 13% of aortic valves removed during valve replacement surgery contain lamellar bone-like organized structures (10).

Many lines of evidence suggest that VC is an actively regulated process in which *in situ* phenotypic transition of VICs into osteoblast-like cells and myofibroblasts occurs (8, 11, 12). Studies indicated that excessive formation of reactive oxygen species (ROS) play a crucial role in the initiation and progression of these processes (13). The osteogenic transition of VICs is characterized by elevated expression of osteogenic markers including runt-related transcription factor 2 (Runx2), bone morphogenetic protein 2 (BMP2), alkaline phosphatase (ALP), osteopontin (OPN) and osteocalcin (OCN) (14, 15). Importantly, these osteogenic markers are found to be upregulated along with increased ROS production in calcified human aortic valves (14–16).

Most of the healthy human heart valves are avascular, therefore adequate nutrition and oxygenation of VICs are ensured via diffusion from the circulating blood (17–19), [reviewed in (20)]. On the other hand, valve thickening compromise the diffusional oxygen transfer, and additional blood supply is required to support the needs of active metabolism of valve cells. In line of this notion, a large body of evidence show the presence of intrinsic neovasculature in thickened and stenotic valves (19, 21), [reviewed in (20)]. Formation of neovessels is found to be associated with increased expression of hypoxia inducible factor (HIF) alpha subunits HIF-1*α* and HIF-2*α* (22, 23), activation of the HIF pathway and upregulation of vascular endothelial growth factor (20, 22, 24–28). Additionally, studies revealed that neovessel density correlates with valve calcification (19–22, 24).

Hypoxia and sustained HIF activation have been shown to promote vascular smooth muscle cells (VSMCs) phenotype switch towards osteoblast-like cells, and accelerate vascular calcification (29–31). Therefore, in this work we have investigated whether hypoxia and HIF signaling are actively participating in osteogenic trans-differentiation of VICs and subsequent VC. We choose the adenine and high phosphate-induced CKD model as our *in vivo* approach and high inorganic phosphate (Pi)-induced calcification of human VICs for the *in vitro* experiments.

## 2. Materials and methods

### 2.1. Materials

We purchased all the reagents from Sigma-Aldrich Co (St. Louis, MO, USA) unless indicated otherwise.

### 2.2. Induction of CKD and DPD treatment in mice

Mice were kept in plastic cages with standard beddings in 12-hour light—12 h dark cycles and unlimited access to food and water. We performed the experiments with the approval of the Institutional Ethics Committee of University of Debrecen under a registration number of 10/2021/DEMÁB, and all procedures conformed to the guidelines from Directive 2010/63/EU of the European Parliament on the protection of animals used for scientific purposes. Animal studies were reported in compliance with the ARRIVE guidelines.

Ten male C57BL/6 mice (8–10 weeks old, *n* = 5/group) were randomly divided into 2 groups: control (Ctrl) and CKD. CKD was induced by an adenine-containing diet as described previously (31, 32). In the first 6 weeks the mice received a diet containing adenine (0.2%) and elevated phosphate (0.7%) followed by adenine (0.2%) and high phosphate (1.8%) diet (S8106-S075 and S8893-S006 respectively, Ssniff, Soest, Germany) for 4 weeks.

In a separate experiment we tested the effect of the hypoxia mimetic drug Daprodustat DPD (HY-17608, MedChemExpress, NJ, USA) on calcification. To this end, 15 male C57BL/6 mice (8–10 weeks old) were divided into 3 groups (Ctrl, CKD, CKD + DPD, *n* = 5/group). DPD was suspended in 1% methylcellulose and was administered orally at a dose of 15 mg/kg/day between weeks 7 and 10 as described previously (31). The dose of DPD is the minimal dose that corrects anemia in C57BL/6 mice which was chosen based on our previous study (31). We euthanized the mice by CO_2_ inhalation at the end of the experiments, and collected blood by cardiac puncture for analysis.

### 2.3. Laboratory analysis of renal function and anemia in mice

Plasma phosphate, urea and creatinine levels were assessed spectrophotometrically and by a kinetic assay respectively, on a Cobas^R^ 6,000 device (Roche Diagnostics, Mannheim, Germany). Hematology parameters were determined from citrate-anticoagulated whole blood by a Siemens Advia-2120i analyzer (Siemens, Tarrytown, NY, USA) with the use of 800 Mouse C57BL program of Multi Species software.

### 2.4. Imaging and quantification of aortic calcification

OsteoSense™ dye (OsteoSense 680 EX and NEV10020EX; PerkinElmer, MA, USA) was reconstituted in DPBS in a concentration of 20 nmol/ml. We anesthetized the mice with isoflurane inhalation and injected the dye in a dose of 2 nmol/20 g body weight through the retro-orbital venous sinus. Imaging was performed 24 h post-injection. We euthanized the mice with CO_2_ inhalation, perfused with 5 ml of PBS, and analyzed the isolated hearts ex vivo by an IVIS Spectrum In Vivo Imaging System (PerkinElmer, MA, USA).

### 2.5. Histology and immunohistochemistry

After the OsteoSense™ imaging, the isolated hearts were fixed in 10% neutral buffered formalin and were embedded in paraffin blocks and cut into 4–5 µm-thick cross-sections. Sections were deparaffinized and rehydrated followed by von Kossa and Alizarin Red stainings with standard procedures. All the sections were counterstained with hematoxylin eosin. Von Kossa staining was quantified by Image J software.

### 2.6. Cell culture and reagents

Human VICs (P10462, Innoprot, Bizkaia, Spain) were maintained in Fibroblast Medium (P60108, Innoprot) supplemented with 10% FBS (10270-106, Gibco, Grand Island, NY, USA), sodium pyruvate, L-glutamine and antibiotic antimycotic solution, according to the manufacturer's protocol. Cells were cultured at 37°C in a humidified atmosphere with 5% CO_2_ content. We performed the experiments on VICs derived from 3 different donors between passages 4 and 8.

To induce calcification we exposed VICs to an osteogenic medium (OM) which was obtained by supplementing the growth medium with inorganic phosphate (Pi in the form of NaH_2_PO_4_ and Na_2_HPO_4_, pH 7.4, 2.5 mmol/L, or as indicated) and Ca (CaCl_2_, 0.3 mmol/L). Both growth medium and OM were changed in every other day throughout the experiments.

### 2.7. Hypoxic treatment

To provide hypoxic environment we placed the cells into a modular incubator chamber (Billups-Rothenberg Inc, Del Mar, CA, USA). We filled the chamber with a gas mixture of 1% O_2_, 5% CO_2_, and 94% of N_2_ (Linde, Dublin, Ireland) and applied a continuous slow flow (0.1 L/min) of the gas throughout the experiment. For normoxia, we used a gas mixture of 21% O_2_, 5% CO_2_, and 74% of N_2_. In other experiments, we used hypoxia mimetic drugs such as desferrioxamine (DFO, 40 *μ*mol/L), CoCl_2_ (200 *μ*mol/L) and DPD (20 µmol/L) or the HIF-1 inhibitor chetomin (Tocris, Bristol, United Kingdom, 12 nmol/L).

### 2.8. Alizarin red staining and quantification

At the end of the experiment we washed the cells with PBS, and fixed with 4% paraformaldehyde for 20 min. After rinsing with PBS we stained the cells with Alizarin Red S solution (2%, pH 4.2) for 10 min at room temperature. Following this we applied several washes with deionized water to remove unbound dye. After taking pictures of the staining, we dissolved the dye in 100 µl of 100 mmol/L hexadecylpyridinium-chloride and determined optical density at 560 nm. Experiments were repeated at least three times minimum in triplicates.

### 2.9. Quantification of Ca deposition

VICs cultured in 96-well plates were washed with PBS and decalcified with HCl for 30 min at room temperature. We measured Ca content from HCl-containing supernatants with QuantiChrom Calcium Assay kit (Gentaur, Kampenhout, Belgium). To obtain protein concentration, we washed the cells with PBS and lysed in a lysis buffer containing NaOH (0.1 mol/L) and sodium dodecyl sulphate (0.1%). We determined protein concentration with BCA protein assay kit (ThermoFisher, Waltham, MA, USA) and nomalized Ca content of the cells to protein content. Experiments were repeated at least three times in triplicates.

### 2.10. Quantification of OCN

VICs were cultured in 6-well plates. After removing the medium, we added 100 *μ*l of EDTA (0.5 mol/L, pH 6.9) to the wells. We quantified OCN content of the EDTA-solubilized samples by an enzyme-linked immunosorbent assay (Bio-Techne R&D Systems, Minneapolis, MN, USA). OCN content was normalized to protein content and expressed as ng OCN/mg protein. Experiments were repeated at least three times in duplicates.

### 2.11. Real-time qPCR

RNA was isolated from the hearts of the mice with Tri reagent (Molecular Research Center, Cincinnati, OH, USA) according to the manufacturer's protocol. To prepare cDNA we used High Capacity cDNA Reverse Transcription Kit (Applied Biosystems, Waltham, USA). The qPCR was carried out on a BioRad CFX96 Real-time System (Bio-Rad, Hercules, CA, USA) with the use of iTaq™ Universal SYBR® Green Supermix (Bio-Rad) and predesigned primers to detect mRNA levels of HIF-1*α*, HIF-2*α*, Runx2 and Sox9 (Table 1). We used the comparative Ct method to calculate the expression level of the transcripts, and mouse HPRT was used for normalization as internal control. Experiments were repeated at least three times in triplicates.

**Table 1:** List of primers used in quantitative PCR.

Gene	Forward	Reverse
HIF-1*α*	5′-GTTGCCACTTCCCCACAATG-3’	5′-TTCACTGTCTAGACCACCGG-3′
HIF-2α	5′-TCGGACACATAAGCTCCTGT-3′	5′-CCACAGCAATGAAACCCTCC-3′
Runx2	5′-GCATCCTATCAGTTCCCAATG-3′	5′-GAGGTGGTGGTGCATGGT-3′
Sox9	5′-GCTCTACTCCACCTTCACTTAC-3′	5′-TGTGTGTAGACTGGTTGTTCC-3′
HPRT	5′- TCCTCCTCAGACCGCTTTT-3′	5′- CCTGGTTCATCATCGCTAATC-3′

### 2.12. Western blot analysis

We lysed VICs in Laemmli lysis buffer and the cell lysate was resolved by SDS-PAGE (7.5%–10%). Proteins were blotted onto nitrocellulose membranes (Amersham, GE Healthcare, Chicago, IL, USA). Western blotting was performed with the use of primary antibodies listed in Table 2. Secondary antibodies—horseradish peroxidase linked rabbit (NA-934) and mouse IgG (NA-931) (Amersham)—were applied at a concentration of 0.5 µg/ml. Blots were developed with enhanced chemiluminescence system Clarity Western ECL (BioRad, Hercules, CA, USA). Chemiluminescent signals were either detected on an x-ray film or with a C-Digit Blot Scanner (LI-COR Biosciences, Lincoln, NE, USA). Following the development, all membranes were stripped and re-probed for *β*-actin using anti-*β*-actin antibody at a concentration of 0.5 µg/ml (sc-47778, Santa Cruz Biotechnology Inc., Dallas, TX, USA). We used the inbuilt software of the C-Digit Blot Scanner for quantification. Experiments were repeated three times.

**Table 2:** List of primary antibodies used in western blot.

Protein	Company, catalog number	Concentration
HIF-1α	GeneTex (Irvine, CA, USA), GTX127309	1 µg/ml
HIF-2α	Cell Signaling (Danvers, Massachusetts, USA), #7096	3 µg/ml
Glut-1	GeneTex (Irvine, CA, USA), GTX15309	0.25 µg/ml
Runx2	Proteintech (Rosemont, IL, USA), 20700-1-AP	0.6 µg/ml
Sox9	Invitrogen (Carlsbad, CA, USA), PA5-81966	0.1 ug/ml
ALP	Santa Cruz Biotech. Inc. (Dallas, TX, USA), sc-365765	0.4 µg/ml

### 2.13. RNA silencing

We used Lipofectamine RNAiMAX reagent (Invitrogen, Carlsbad, CA, USA) to transfect VICs with siRNA. We followed the protocol that was provided by the manufacturer. The siRNA for HIF-1*α* (AM16708, ID: 106498) and HIF-2*α* (AM16708, ID: 106446) and silencer negative control #1 (4390843) were purchased from Invitrogen. To confirm the efficiency of silencing we performed Western blot analysis. Experiments were repeated at least three times.

### 2.14. Intracellular ROS measurement

The level of ROS was measured with CM-H2DCFDA assay (Life Technologies, Carlsbad, CA, USA). The cells were loaded with the dye (10 *μ*mol/L, 30 min), then washed thoroughly with HBSS. After a 4-hour treatment the cells were washed with HBSS and the fluorescence intensity was evaluated with the use of 488 nm excitation and 533 nm emission wavelengths. In some experiments, we applied the ROS inhibitor N-acetyl cysteine (NAC, 1 mmol/L) during the treatment. Experiments were repeated at least three times in quadruplicates.

### 2.15. Determination of cell viability

We performed an MTT assay to measure cell viability. A solution of 3-[4, 5-Dimethylthiazol-2-yl]-2,5-diphenyl-tetrazolium bromide (0.5 mg/mL in HBSS) was incubated with the cells for 4 h. Following this, we removed the MTT solution and dissolved the formazan crystals in 100 *μ*l of DMSO. Using DMSO as a blank, we measured optical density of the samples at 570 nm. Experiments were repeated at least three times in quadruplicates.

### 2.16. Data analysis

We show all the results as mean ± SD. We used GraphPad Prism software (version 8.01, San Diego, CA, USA) to perform statistical analyses. Normality of distribution was assessed by Shapiro-Wilk test. All data passed normality and equal variance tests, therefore we used parametric tests to determine *p* values. Two-tailed Student's t-test (in case of two groups) and one-way ANOVA followed by Tukey's *post hoc* test (in case of more than two groups) were used to determine statistically significant differences between the groups. A value of p < 0.05 was considered significant.

## 3. Results

### 3.1. Activation of osteogenic and hypoxia signaling in heart of CKD mice and in valve interstitial cells (VICs) exposed to high phosphate

**Figure 1:**
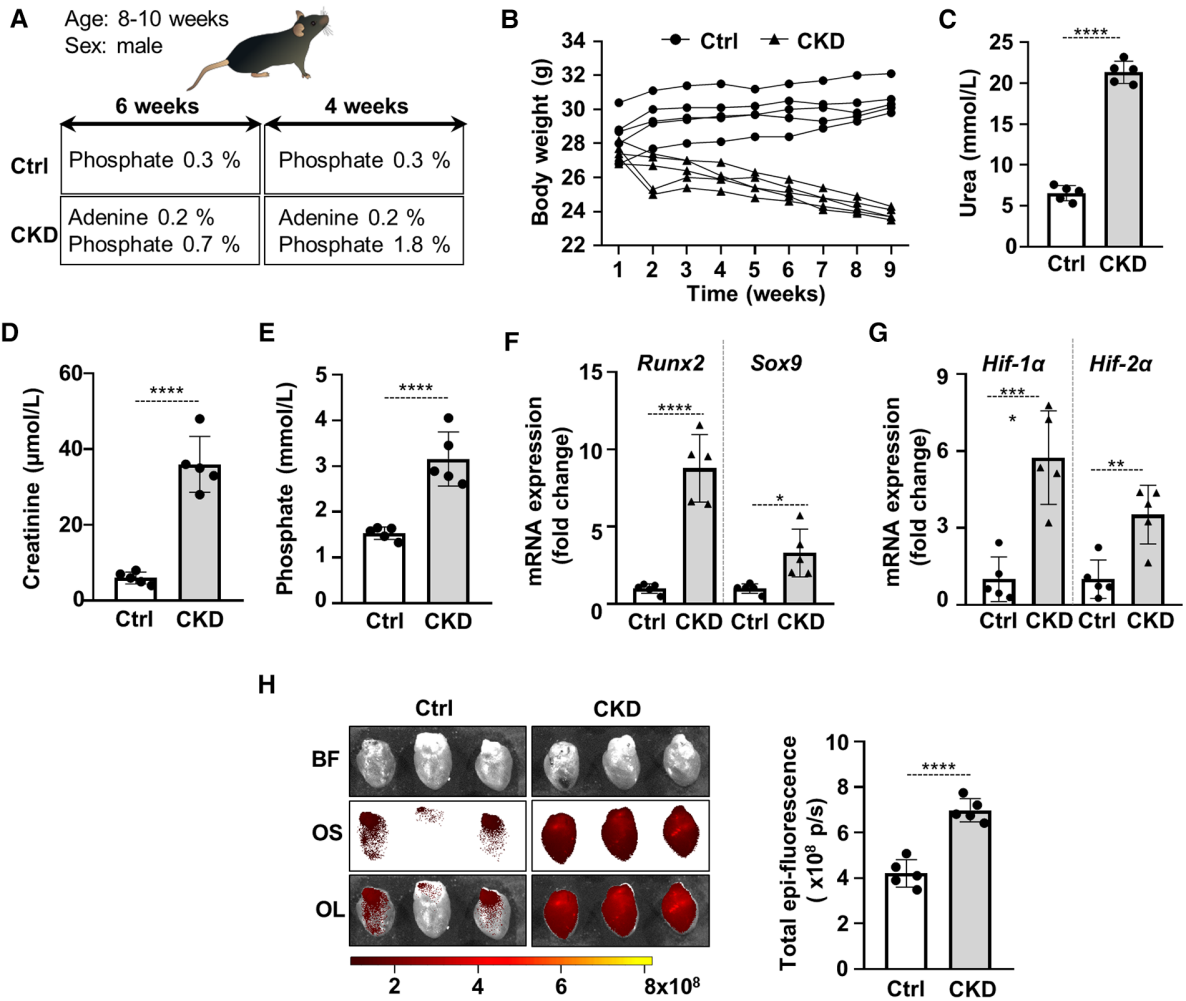
Activation of osteogenic and hypoxia signaling and calcification in the heart of CKD mice. (**A**) Scheme of the experimental protocol. (**B**) Body weight, (**C**) plasma urea, (**D**) plasma creatinine, (**E**) plasma phosphate levels in control (Ctrl) and CKD mice (*n* = 5/group). (**F,G**) Relative mRNA expressions of Runx2, Sox9, HIF-1*α* and HIF-1*α* normalized to HPRT from heart tissue derived from Ctrl and CKD mice (*n* = 5, measured in triplicates). (**H**) Bright-field and macroscopic fluorescence reflectance imaging of calcification and quantification in the heart of Ctrl and CKD mice (*n* = 5/group). Data are expressed as mean ± SD. Ordinary one-way ANOVA followed by Tukey's multiply comparison test was used to calculate *p* values. *p < 0.05, **p < 0.01, ***p < 0.005, ****p < 0.001.

Cardiac VC is the main cause of cardiovascular disease and mortality in CKD patients. We induced CKD in C57BL/6 mice with a two-phase diet containing adenine (0.2%) and moderately elevated phosphate (0.7%) in the first 6 weeks and adenine (0.2%) and high phosphate (1.8%) in the following 4 weeks. Control mice (Ctrl) received a standard mice diet with 0.3% phosphate content (Figure 1A). The development of CKD was associated with significant decrease in body weight (Figure 1B), and increased urea, creatinine and phosphate levels in plasma (Figures 1C–E). To address whether CKD induces osteogenic and hypoxia pathways, we determined mRNA levels of osteogenic transcription factors Runx2 and Sox9 and hypoxia markers HIF-1*α* and HIF-2*α* in the heart of Ctrl and CKD mice. Both osteogenic and hypoxia markers were elevated in the heart tissue of CKD mice in comparison to Ctrl (Figures 1F,G). Furthermore, to evaluate osteogenic activity in mouse hearts we performed OsteoSense™ staining in Ctrl and CKD mice. Fluorescent intensity of the heart tissue was higher in CKD mice compared to Ctrl mice (4.21 × 10^8^ vs. 6.99 × 10^8 ^p/s, *p* < 0.001, Figure 1H).

**Figure 2:**
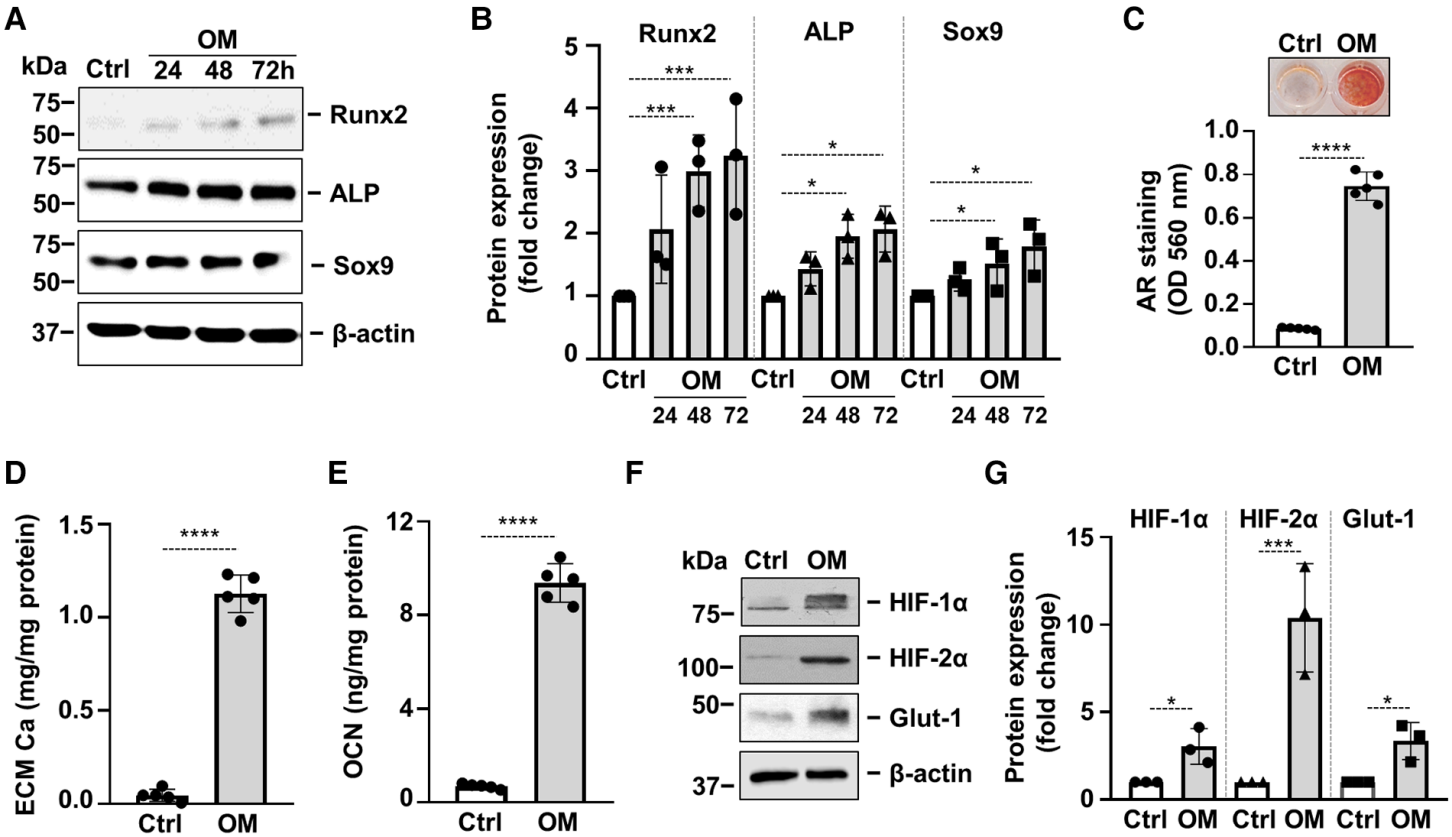
Osteogenic stimulation induces osteogenic transdifferentiation, calcification and activation of hypoxia signaling in VICs. Confluent VICs were cultured in Ctrl or osteogenic conditions (OM, 2.5 mmol/L excess Pi, 0.3 mmol/L excess Ca over Ctrl). (**A,B**) Runx2, ALP and Sox9 protein expressions detected by Western Blot from whole cell lysate (24, 48, 72 h). Membranes were re-probed for *β*-actin. Representative Western blots and densitometry analysis from three independent experiments. (**C**) Calcium deposition in the ECM (day 5) evaluated by AR staining. Representative image and quantification are depicted from 5 independent experiments. (**D**) Calcium content of the HCl-solubilized ECM. (**E**) OCN level of EDTA-solubilized ECM (day 10). (**F,G**) Protein expression of HIF-1*α* and HIF-2*α* in whole cell lysates (24 h). Membranes were re-probed for *β*-actin. Representative Western blots and relative expression of HIF-1*α* and HIF-2*α* normalized to *β*-actin from 3 independent experiments. (**E,G**) Representative AR staining (day 4) and quantification. Data are expressed as mean ± SD. Ordinary one-way ANOVA followed by Tukey's multiply comparison test was used to calculate *p* values. *p < 0.05, **p < 0.01, ***p < 0.005, ****p < 0.001.

Osteogenic trans-differentiation and extracellular matrix (ECM) mineralization of VICs play a major role in the development of cardiac VC. To set up an *in vitro* model of VC we treated VICs with osteogenic medium (OM: growth medium supplemented with 2.5 mmol/L Pi and 0.3 mmol/L Ca). In response to OM we observed time-dependent upregulation of Runx2 and Sox9, the master transcription factors regulating osteogenesis and chondrogenesis respectively, as well as alkaline phosphatase (ALP) (Figures 2A,B). OM triggered calcification of VICs which was assessed by Alizarin Red staining and Ca measurement from HCl-solubilized ECM (Figures 2C,D). Furthermore, OM induced deposition of the Ca-binding protein osteocalcin (OCN) in the ECM (Figure 2E). Along with these responses, OM also triggered a hypoxia response in VICs, characterized by elevated protein expression of HIF-1*α*, HIF-2*α* and Glut-1 (Figures 2F,G).

### 3.2. Hypoxia signaling is involved in high Pi-induced calcification of VICs

Recent works highlighted that hypoxia signaling is activated in calcifying aorta and showed that hypoxia inducible factors (HIFs) play a critical role in osteogenic differentiation of VSMCs (26–28).

**Figure 3:**
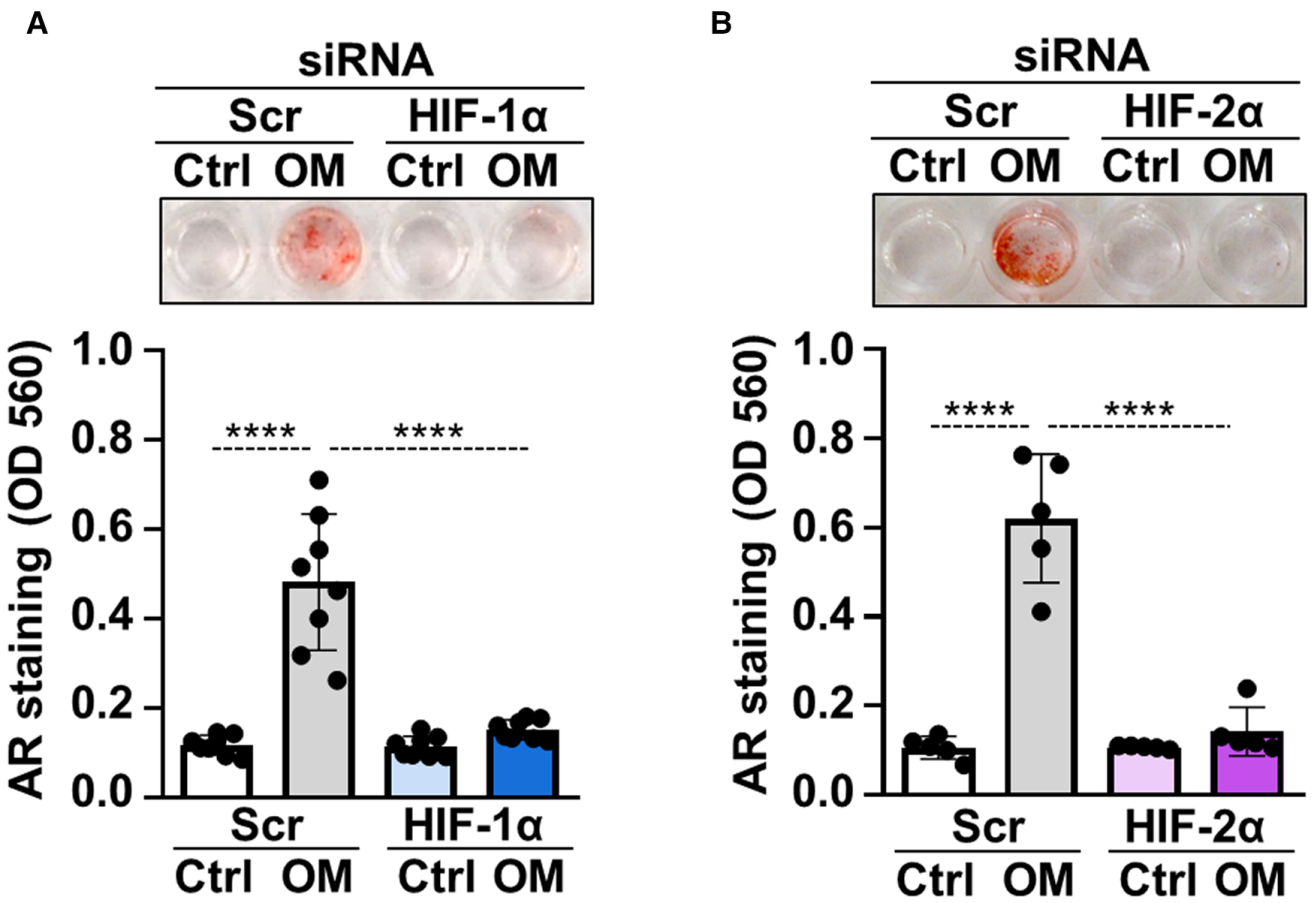
HIF pathway is critically involved in osteogenic trans-differentiation of VICs. (**A,B**) Confluent VICs were cultured in control (Ctrl) or osteogenic conditions (OM, 2.5 mmol/L excess Pi, 0.3 mmol/L excess Ca over Ctrl) in the presence of HIF-1*α*, HIF-2*α* or scrambled siRNA. Representative AR staining (day 4) and quantification. Data are expressed as mean ± SD. Ordinary one-way ANOVA followed by Tukey's multiply comparison test was used to calculate *p* values. ****p < 0.001.

To address whether hypoxia signaling is implicated in osteogenic differentiation of VICs, we used siRNA to downregulate protein expressions of HIF-1*α* and HIF-2*α*, the regulatory subunits of the HIF complexes. Western blots revealed that the gene silencing approaches were successful (Supplementary Figures S1A,B). Knockdown of either HIF-1*α* or HIF-2*α* was associated with decreased calcification of VICs as assessed by Alizarin Red staining (Figures 3A,B) suggesting that HIF pathways are not only activated upon osteogenic stimulation, but they are actively participated in the calcification process.

### 3.3. Hypoxia enhances calcification of VICs in a HIF-1*α*- and HIF-2*α*-dependent manner

**Figure 4:**
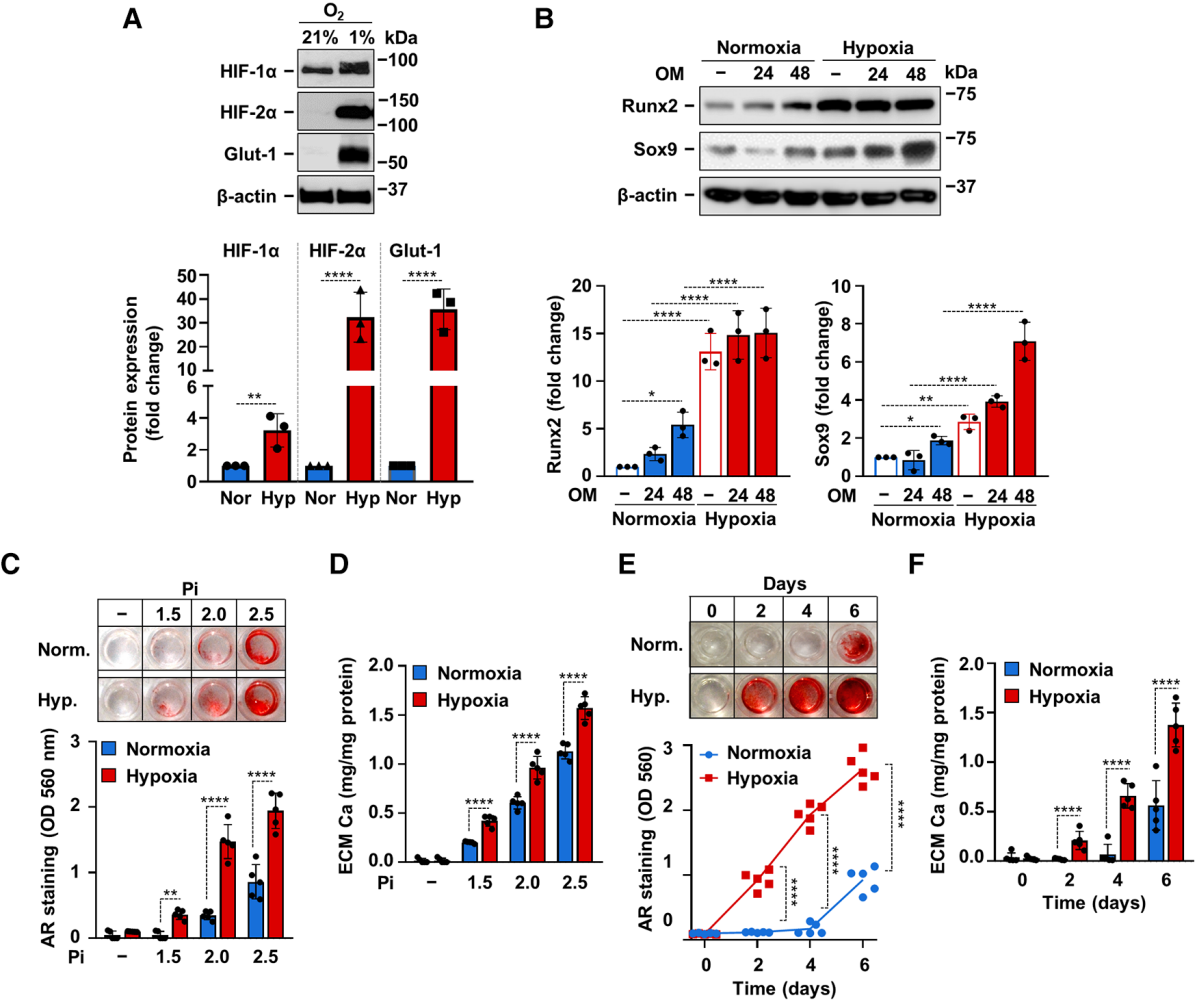
Hypoxia enhances OM-induced calcification of VICs. (**A**) Confluent VICs were maintained under normoxic (Nor, 21% O_2_) or hypoxic (Hyp, 1% O_2_) conditions. (**A**) HIF-1*α*, HIF-2*α*, Glut-1 and *β*-actin protein expressions detected by Western Blot from whole cell lysate (24 h). Representative Western blots and densitometry analysis from three independent experiments. (**B**) Confluent VICs under normoxic (21% O_2_) or hypoxic (1% O_2_) conditions were exposed to OM (2.5 mmol/L excess Pi, 0.3 mmol/L excess Ca over Ctrl). Runx2 and Sox9 protein expressions detected by Western Blot from whole cell lysate (24, 48 h). Membranes were re-probed for *β*-actin. Representative Western blots and densitometry analysis from three independent experiments. (**C,D**) Confluent VICs were exposed to OM with different Pi content (1.5–2.5 mmol/L excess over Ctrl) under normoxic (21% O_2_) and hypoxic conditions (1% O_2_). (**C**) Representative AR staining (day 6) and quantification. (**D**) Calcium content of the HCl-solubilized ECM (day 6). (**E,F**) Time course of calcium accumulation under normoxic and hypoxic conditions in the presence of OM. (**E**) Representative AR staining and quantification. (**F**) Calcium content of the HCl-solubilized ECM. Data are expressed as mean ± SD. (**A-D,F**) Ordinary one-way ANOVA followed by Tukey's multiply comparison test was used to obtain *p* values. (**E**) Multiply t-tests to compare normoxia and hypoxia samples at each time points were performed to obtain *p* values. *p < 0.05, **p < 0.01, ****p < 0.001.

After defining the crucial involvement of hypoxia signaling in phosphate-induced calcification of VICs we asked whether hypoxia influences OM-induced osteogenic differentiation and calcification of VICs. First, we exposed VICs to normoxia (21% O_2_) or hypoxia (1% O_2_) for 24 h and evaluated protein expressions of HIF-1*α*, HIF-2*α* and Glut-1. As expected, hypoxia triggered a hypoxia response in VICs characterized by elevated protein expression of HIF-1*α*, HIF-2*α* and Glut-1 (Figure 4A). Then we treated VICs with OM (2.5 mmol/L Pi, 0.3 mmol/L Ca) under normoxic (21% O_2_) and hypoxic (1% O_2_) conditions for 24 and 48 h. Compared to control, OM slightly increased Runx2 and Sox9 expressions under normoxic condition after 48 h of exposure (Figure 4B). On the other hand, hypoxia strongly upregulated Runx2 expression even in the absence of OM stimulation (Figure 4B). Osteogenic stimuli could not further increase Runx2 expression under hypoxia (Figure 4B). Compared to normoxia, Sox9 expression was elevated under hypoxia at each condition (Figure 4B). These results suggest that hypoxia may exaggerate osteogenic reprogramming of VICs.

Next, we addressed the effect of hypoxia on ECM calcification in VICs. We induced VICs calcification with OM containing calcium (0.3 mmol/L excess) and different amounts of excess Pi (1.5; 2.0; 2.5 mmol/L) under normoxic and hypoxic conditions. As revealed by Alizarin Red staining and calcium measurement, hypoxia potentiated the pro-calcification effect of Pi at each tested concentrations (Figures 4C,D). Then we investigated time-dependency of VICs calcification under normoxic and hypoxic conditions. Alizarin Red staining showed positivity after 2 days of OM exposure under hypoxic condition, whereas calcification became detectable only on day 6 under normoxia (Figure 4E). Calcium measurement from HCl-solubilized ECM also supported the finding that hypoxia potentiates and accelerates Pi-induced calcification of VICs (Figure 4F).

**Figure 5:**
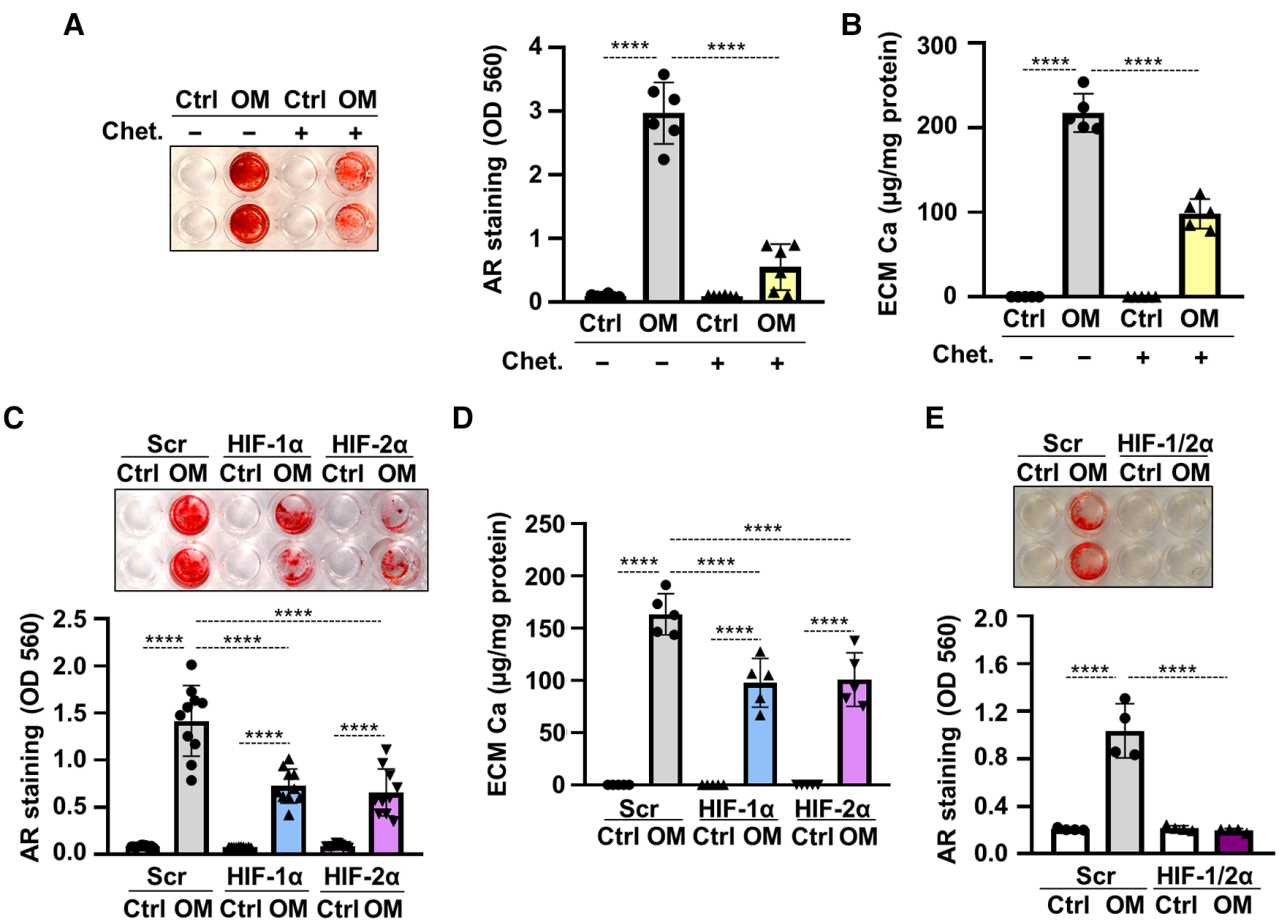
Hypoxia enhances OM-induced osteogenic trans-differentiation of VICs through HIF-1 signaling. (**A,B**) Confluent VICs were maintained in Ctrl or OM (2.5 mmol/L excess Pi, 0.3 mmol/L excess Ca over Ctrl) conditions under hypoxia (1% O_2_) in the presence or absence of the HIF-1 inhibitor chetomin (Chet, 12 nmol/L). (**A**) Representative AR staining (day 4) and quantification. (**B**) Calcium content of the HCl-solubilized ECM (day 4). (**C–F**) VICs were kept under Ctrl or OM conditions in hypoxia (1% O_2_) in the presence of HIF-1*α*, HIF-2*α* or both, or scrambled siRNA. (**C**) Representative AR staining (day 4) and quantification. (**D**) Calcium content of the HCl-solubilized ECM (day 4). (**E**) Representative AR staining (day 4) and quantification of HIF-1*α*, HIF-2*α* double knocked-down cells. Data are expressed as mean ± SD. *p* values were calculated using one-way ANOVA followed by Tukey's multiply comparison analysis. ****p < 0.001.

To see whether HIF signaling was involved in hypoxia-induced acceleration of VICs calcification, first we applied the HIF inhibitor chetomin and investigated OM-induced calcification under hypoxic condition. As shown by Alizarin Red staining and calcium measurement, chetomin inhibited calcification of VICs (Figures 5A,B). Then we knocked-down HIF-1*α*, HIF-2*α* or both with the use of target-specific siRNAs under hypoxia. Western blots revealed that the gene silencing approaches were successful (Supplementary Figures S1C,D). Silencing of either HIF-1*α* or HIF-2*α* resulted in attenuation, whereas silencing of both HIF-α subunits caused complete inhibition of hypoxia-induced calcification (Figures 5C–E), supporting the involvement of HIF signaling in hypoxia-induced VICs calcification.

### 3.4. The involvement of ROS in hypoxia-mediated potentiation of VICs calcification

**Figure 6:**
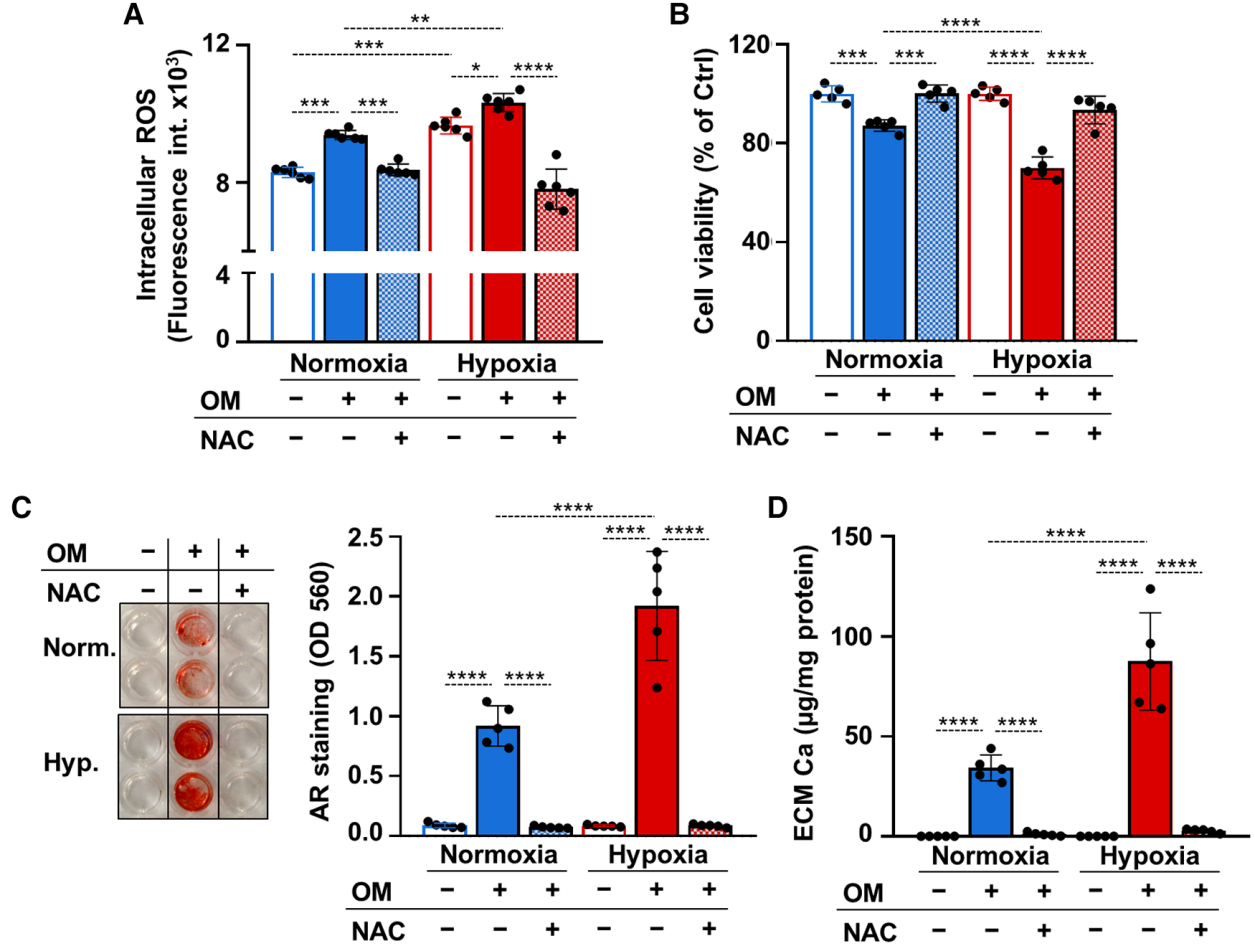
ROS regulate calcification of VICs under both normoxia and hypoxia. (**A–D**) Confluent VICs were maintained under normoxia (21% O_2_) or hypoxia (1% O_2_) in Ctrl or OM conditions in the presence or absence of NAC (1 mmol/L). (**A**) Intracellular ROS production in VICs after a 4-hour exposure. (**B**) Cell viability assessed by MTT assay after 4 days of exposure. (**C**) Representative AR staining (day 4) and quantification. (**B**) Calcium content of the HCl-solubilized ECM (day 4). Data are expressed as mean ± SD. Ordinary one-way ANOVA followed by Tukey's multiply comparison test was used to obtain *p* values. *p < 0.05, **p < 0.01, ***p < 0.005, ****p < 0.001.

Recent evidence suggested a causative role for excess ROS-mediated oxidative stress in the osteogenic differentiation of VICs (13, 16). To explore whether unfettered production of ROS is implicated in VICs calcification under hypoxia we measured ROS production in control and OM-stimulated VICs under normoxic and hypoxic conditions. Osteogenic stimulation increased ROS production under normoxia (Figure 6A). Compared to normoxia, hypoxia increased ROS production in VICs in both control and OM conditions (Figure 6A). The glutathione precursor, N-acetyl-cysteine (NAC) attenuated excessive ROS production in all conditions (Figure 6A).

Apoptotic cell death and the release of apoptotic bodies is an important calcification mechanism. Excess ROS production can trigger cell death, therefore next we investigated cell viability in control and OM-treated VICs under normoxia and hypoxia after 4 days of exposure in the presence or absence of NAC. Osteogenic stimulation triggered a decline in cell viability in normoxia and even more cell death was observed in hypoxia (Figure 6B). NAC prevented OM-induced cell death under both normoxia and hypoxia (Figure 6B). Attenuation of unfettered ROS production and cell death by NAC was associated with complete inhibition of OM-induced VICs calcification as revealed by Alizarin red staining and calcium measurements (Figures 6C,D).

### 3.5. Hypoxia mimetic drugs enhance VICs calcification

**Figure 7:**
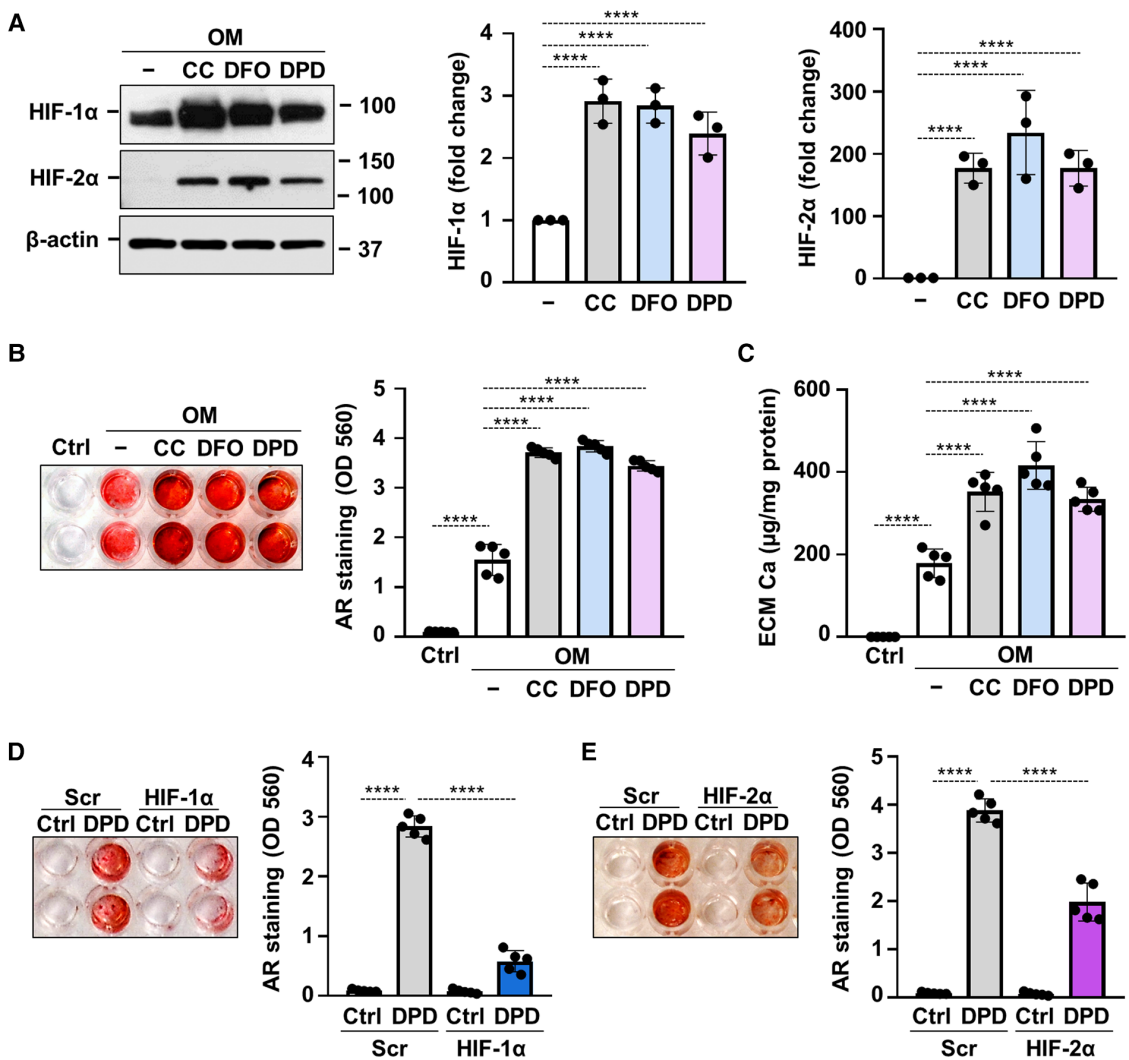
Hypoxia mimetic drugs augment OM-induced calcification of VICs. (**A–C**) Confluent VICs maintained in OM (2.5 mmol/L excess Pi, 0.3 mmol/L excess Ca) were treated with hypoxia mimetic drugs CoCl_2_ (CC, 200 µmol/L), desferrioxamine (DFO, 40 µmol/L) and Daprodustat (DPD, 20 µmol/L). (**A**) Protein expressions of HIF-1*α* and HIF-2*α* were detected by Western Blot in whole cell lysates (24 h). Membranes were re-probed for *β*-actin. Representative Western blots and densitometry analysis from three independent experiments. (**B**) Representative AR staining (day 5) and quantification. (**C**) Calcium content of the HCl-solubilized ECM (day 5). (**D,E**) VICs were kept under Ctrl or OM + DPD conditions in the presence of HIF-1*α* or HIF-2*α* or scrambled siRNA. Representative AR staining (day 4) and quantification. Data are expressed as mean ± SD. Ordinary one-way ANOVA followed by Tukey's multiply comparison test was used to obtain *p* values. *p < 0.05, **p < 0.01, ***p < 0.005, ****p < 0.001.

Hypoxia mimetic drugs mimic the effect of real hypoxia through the stabilization of HIF*α* subunits. We investigated three different hypoxia mimetic drugs, cobalt-chloride (CoCl_2_), desferrioxamine (DFO) and Daprodustat (DPD), to see whether they influence Pi-induced VICs calcification under normoxic condition. We treated VICs with CoCl_2_ (200 µmol/L), DFO (40 µmol/L) or DPD (20 µmol/L) for 24 h and first we evaluated protein expressions of HIF-1*α* and HIF-2*α* from whole cell lysate (Figure 7A). Hypoxia mimetics increased both HIF-1*α* and HIF-2*α* levels markedly in VICs.

Next, we investigated the effects of hypoxia mimetic drugs on OM-induced calcification of VICs. We treated VICs with OM (0.3 mmol/L excess Ca, 2.5 mmol/L excess Pi) in the presence or absence of CoCl_2_ (200 µmol/L), DFO (40 µmol/L) or DPD (20 µmol/L). Alizarin Red staining and calcium measurement were performed on day 5. We observed that all the three tested hypoxia mimetic drugs enhanced OM-induced calcification in VICs (Figures 7B,C). These results suggest that not only real hypoxia but also chemical activation of the HIF pathways enhances calcification of VICs.

Silencing of either HIF-1*α* or HIF-2*α* resulted in partial inhibition of OM + DPD-induced calcification as assessed by Alizarin Red staining (Figures 7D,E), pointing out the contribution of HIF signaling to the promotion of VIC calcification by DPD.

### 3.6. DPD enhances aortic VC in CKD mice

**Figure 8:**
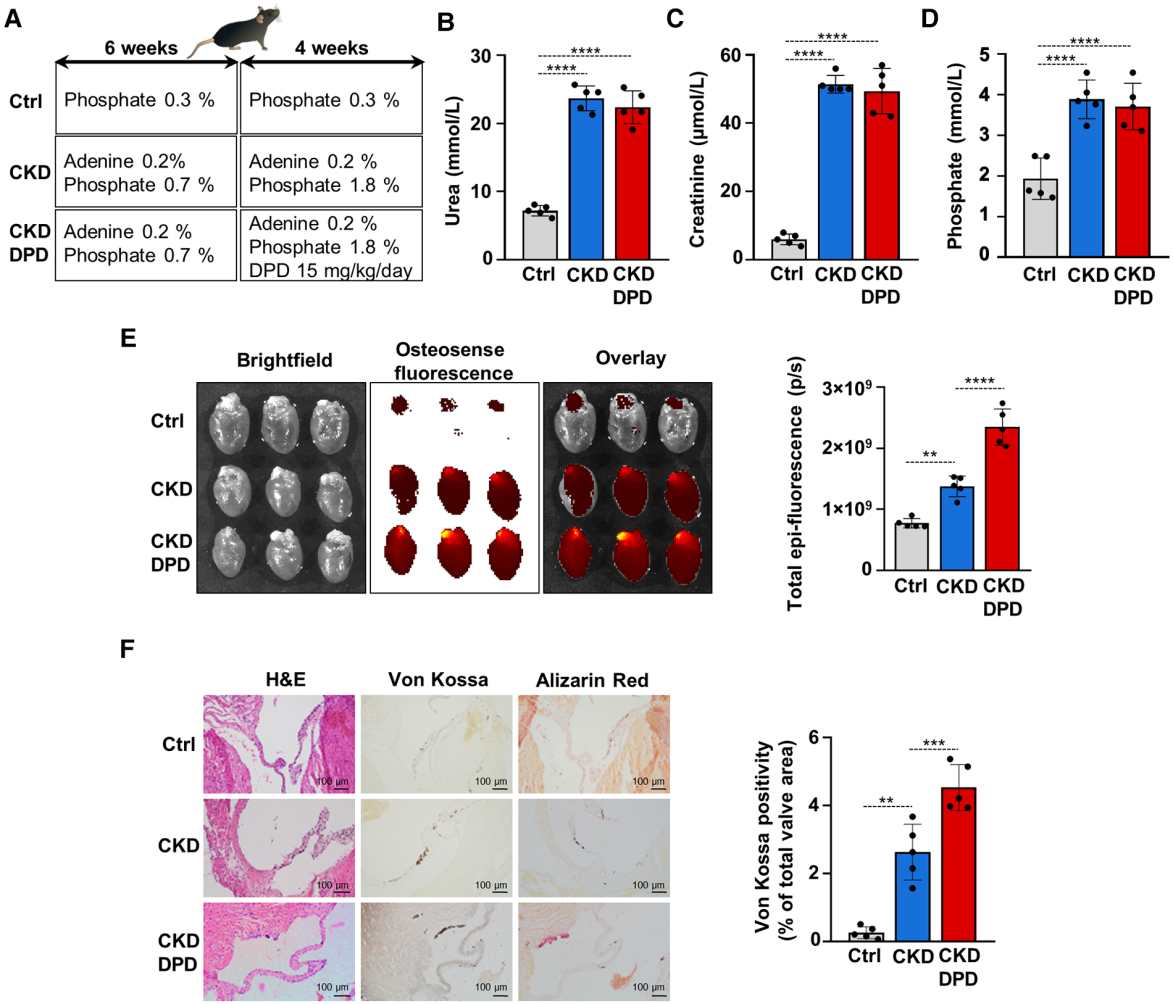
DPD increases aortic VC in mice with CKD. (**A**) Scheme of the experimental protocol. (**B**) Plasma urea, (**C**) creatinine, (**D**) phosphate levels (*n *= 5/group). (**E**) Bright-field and macroscopic fluorescence reflectance imaging of calcification and quantification in the heart of Ctrl, CKD and CKD + DPD mice (*n *= 5/group). (**F**) Histological analysis of heart valves obtained from Ctrl, CKD, and CKD + DPD mice. Representative H&E, von Kossa-stained and alizarin red-stained heart sections and quantification of von Kossa staining. Scalebar: 100 µm. Data are expressed as mean ± SD. Ordinary one-way ANOVA followed by Tukey's multiply comparison test was used to obtain *p* values. **p < 0.01, ***p < 0.005, ****p < 0.001.

DPD is a hypoxia mimetic drug that is used to treat anemia in CKD patients in Japan. After seeing that DPD enhances VICs calcification *in vitro* we addressed its effect on VC in the adenine-induced CKD model in male mice. Fifteen C57BL/6 mice (8–10 weeks old, male) were randomly assigned to 3 groups, Ctrl, CKD, and CKD + DPD (Figure 8A). CKD was induced with a diet containing adenine and elevated phosphate (Figure 1A). After 6 weeks, these mice showed signs of deteriorating kidney function characterized by elevated levels of plasma urea, creatinine and phosphate levels (Supplementary Figure S2). Then we increased phosphate content of the diet, and started to administer DPD (15 mg/body weight kg/day orally) in the next 4 weeks of the experiment (Figure 8A). At 10 weeks we terminated the experiment. At this time point, anemia was developed in CKD mice, characterized by reduced Hb concentration, decreased red blood cell count and low hematocrit levels (Table 3). DPD efficiently corrected CKD-associated anemia resulting in normalized Hb concentration, red blood cell count and hematocrit levels, similar to the controls with normal renal function (Table 3). Plasma urea, creatinine and phosphate levels were similarly high in DPD- and vehicle-treated CKD mice (Figures 8B,D). To address the effect of DPD on heart calcification we performed OsteoSense^TM^ staining and detected higher amount of hydroxyapatite deposition in the hearts derived from DPD-treated CKD mice compared to vehicle-treated CKD mice (2.35 × 10^9^ ± 0.3 × 10^9^ vs. 1.38 × 10^9^ ± 0.17 × 10^9 ^p/s, *p* < 0.05) (Figure 8E). Additionally, we performed histological analysis of hearts derived from Ctrl, CKD and CKD + DPD mice to detect VC. We found stronger von Kossa and alizarin red staining in heart valves of CKD + DPD mice compared to CKD, whereas no calcification was detectable in the heart of Ctrl mice (Figure 8F). These results suggest that DPD—at the dose that is efficient to correct CKD-associated anemia -, can accelerate VC in male mice with CKD.

**Table 3:** Hematology parameters.

Hematology parameter	Control	CKD	CKD + DPD	*p* value	*p* value
				Ctrl vs CKD	CKD vs. CKD + DPD
Hemoglobin (g/L)	117.4 ± 3.6	80.2 ± 8.9	119.2 ± 3.3	0.000012	0.0000077
Red blood cell count (T/L)	8.014 ± 0.15	6.044 ± 0.561	8.046 ± 0.29	0.000032	0.000052
Hematocrit	0.439 ± 0.017	0.301 ± 0.031	0.432 ± 0.014	0.000012	0.000013

## 4. Discussion

Our study is the first demonstration that HIF-1 activation is critically implicated in phosphate-induced calcification of VICs. We found elevation of osteogenic markers along with hypoxia markers in the heart tissue of adenine-induced CKD mice, as well as high phosphate-treated VICs. Knock-down of HIF-1*α* or HIF-2*α* resulted attenuation of phosphate-induced calcification of VICs, suggesting a causative role of HIF-1 pathway activation in this process. Further activation of the HIF-1 pathway by either hypoxia or hypoxia mimetics intensified high-phosphate induced calcification of VICs in a HIF-1*α*, HIF-2*α* and ROS-dependent manner. The hypoxia mimetic drug DPD increased osteogenic activity in the heart tissue and intensified aortic valve calcification in adenine-induced male CKD mice.

Previous studies showed that HIF-1*α* along with vascular endothelial growth factor is upregulated in stenotic valves and co-localize with areas of angiogenesis and calcification (20, 22, 24–28). Moreover, neovessel density positively correlates with the extent of valve calcification (19–22, 24). A recent integrated proteomic and metabolomic profile analyses of cardiac valves identified HIF-1 signaling as a key pathway in calcific aortic valve disease (33).

Previous works linked HIF-1 activation and valve calcification. For example, non-hypoxic activation of HIF-1*α* has been shown to play a causative role in lipopolysaccharide and interferon gamma-induced calcification of VICs (34). In a recent work, down-regulation of the HIF1-*α* pathway was found to be responsible for the anti-calcification effect of atractylenolide-1 (35). Similarly to our result (Figure 2), upregulation of HIF-1*α* by high phosphate has been reported in VICs in connection with ferroptosis (36). Our study provided evidence that HIF-1*α* and HIF-2*α* are not only upregulated but taking a regulatory part in the calcification process of VICs (Figure 3). In agreement with our results, the critical involvement of HIF-1*α* activation in high-phosphate-induced calcification of VSMCs has been reported (30).

Tissue hypoxia is implicated in the pathomechanism of many human diseases including kidney disease (37, 38). Hypoxia accelerates the progression of CKD via promoting fibrogenesis of renal fibroblasts, and triggering epithelial-mesenchymal transformation of renal tubular cells (39, 40). Due to CKD-associated anemia and damage of the microvasculature, tissue hypoxia in CKD is not limited to kidney but affects other organs as well (41, 42). In line of this notion, here we showed increased mRNA and protein expression of HIF-1*α* and HIF-2*α* in heart derived from CKD mice (Figure 1).

Surprisingly, despite the growing evidence that VICs are exposed to hypoxia in certain disease conditions the effect of hypoxia on VICs remained mostly undiscovered. Recent studies showed that hypoxia regulates extracellular matrix secretion and induces pathological extracellular remodeling of VICs (43, 44). Additionally, Kanno et al. showed upregulation of several mesenchymal and hematopoietic progenitor markers in VICs under hypoxic (2% O_2_) culture conditions, and connected stemness of hypoxic VICs with increased potential towards osteogenic differentiation (45).

The effect of hypoxia on osteogenic differentiation potential was studied on diverse cells. Similarly to our results presented here (Figures 4,5), hypoxia promoted osteogenic differentiation of VSMCs, multipotent human mesenchymal stromal cells and periosteal cells (30, 46, 47). In contrast, hypoxia has been reported to decrease the expression of osteogenic markers in MG63 osteoblast-like cells (48). According to another study, hypoxia does not influence osteogenic differentiation of primary osteoblasts and mesenchymal precursors, but quick exposure to anoxia inhibits bone nodule formation and calcification through the downregulation of Runx2 (49). Overall, these results suggest that the effect of hypoxia on osteogenic differentiation is finely regulated and cell specific, in which responses the differences in Runx2 promoter activity in osseous and non-osseous cells might play a role (50).

Exacerbated ROS production plays an important causative role in vascular calcification and in the pathophysiology of calcific aortic valve disease (13, 51). Increased ROS production was detected in aortic valve tissue from patients with pathological heart valve dysfunctions in comparison with transplant-derived control tissues (52). The relation between hypoxia and ROS production is controversial, but a majority of the evidence suggests that hypoxia stimulates ROS formation in most types of mammalian cells (53). Hypoxia impairs the function of the mitochondrial electron transport chain complexes leading to increased ROS signals that play critical role in initiating hypoxia response in diverse cell types (54–56). Additionally, a study on pulmonary artery smooth muscle cells revealed that hypoxia-induced mitochondrial ROS activates NADPH oxidases which provides a positive feedback loop of exacerbated ROS formation upon hypoxia (57). Our results revealed that hypoxia increases ROS formation in VICs. Phosphate-induced calcification of VICs was abrogated by the glutathione-precursor NAC under both hypoxic and normoxic conditions, suggesting a causative role of ROS in the phosphate-induced calcification process (Figure 6).

Activation of the HIF pathways takes place through stabilization of the HIF *α* subunits. Normally, HIF *α* subunits are hydroxylated at specific proline residues by prolyl hydroxylase domain proteins (PHDs) and eliminated via the ubiquitin-proteasome degradation pathway (58). Here we showed that non-hypoxic activation of the HIF pathway by PHD inhibitors, cobalt chloride, DFO, and DPD promoted OM-induced calcification of VICs under normoxic condition (Figure 7). In agreement with this result we and others previously showed enhancement of Pi-induced calcification by DPD and Roxadustat in VSMCs under normoxic conditions (31, 59).

CKD is frequently associated with other chronic diseases such as anemia (60). Anemia of patients with advanced CKD was treated with recombinant erythropoietin or erythropoiesis-stimulating agents (ESAs) (61). Unfortunately, safety concerns of ESAs' use have lately been emerged, because studies showed that ESAs increase the risks for major cardiovascular events and accelerate disease progression (61–64).

In this study we used DPD to investigate the effect of HIF-1 pathway activation on valve calcification in the adenine-induced CKD model. The basis of our choice of the experimental model was that DPD is a new-generation drug and approved in Japan since 2020 for the treatment of patients with CKD-associated anemia (65, 66). Here we showed that DPD corrected anemia, but promoted CKD-induced aortic VC *in vivo* (Figure 8). Previously we found similar effect of DPD on aorta calcification (31). Although the clinical relevance of this model is clear, the conclusions are limited to DPD-driven HIF-1 activation. Therefore further studies are needed to investigate the effect of functional hypoxia and other hypoxia mimetic drugs on vascular and aortic valve calcification.

Besides that, our study has further limitations. In our *in vitro* model we used VICs derived from healthy donors and as we do not have access to diseased human valves we could not compare the responses of healthy and calcifying VICs. Additionally, we were not able to obtain VICs from the heart of CKD mice or perform more complete histological analysis of hypoxia response due to the limitation of tissue samples.

Recent phase 3 trials compared the effect of DPD and an injectable ESA in anemic (Hb: 8.0–11.5 g/dl) dialyzed and non-dialyzed patients with CKD (67, 68). These two trials concluded that DPD was non-inferior to ESA with respect to the increase in the Hb level from baseline in both dialysis-dependent and dialysis-independent CKD patients (67, 68). Additionally, they found that the percentages of patients with adverse cardiovascular events were similar in the DPD and ESA groups among CKD patients regardless of dialysis status (67, 68).

In conclusion, here we showed that hypoxic or pharmacological activation of the HIF pathway accelerates phosphate-induced calcification of VICs, in a HIF-1*α*, HIF-2*α* and ROS-dependent manner. The new generation PHD inhibitor DPD increased aortic VC *in vivo* in the adenine-induced murine model of CKD with high plasma phosphate level. Further studies are needed to investigate the potential involvement of this mechanism to the occurrence of major cardiovascular events which was reported to happen in 25.2% of hemodialysis-dependent CKD patients on DPD treatment during a 2.5-year follow-up period, and in 19.5% of non-dialyzed CKD patients on DPD treatment during a 1.9-year follow-up period (67, 68).

## Data Availability

The original contributions presented in the study are included in the article/**Supplementary Material**, further inquiries can be directed to the corresponding author/s.

## References

[1] Ureña-Torres P D’Marco L Raggi P García-Moll X Brandenburg V Mazzaferro S Valvular heart disease and calcification in CKD: more common than appreciated. Nephrol Dial Transplant. (2020) 35:2046–53. 10.1093/NDT/GFZ133 31326992

[2] Marwick TH Amann K Bangalore S Cavalcante JL Charytan DM Craig JC Chronic kidney disease and valvular heart disease: conclusions from a kidney disease: improving global outcomes (KDIGO) controversies conference. Kidney Int. (2019) 96:836–49. 10.1016/J.KINT.2019.06.025 31543156

[3] Kipourou K O’Driscoll JM Sharma R. Valvular heart disease in patients with chronic kidney disease. Eur Cardiol. (2022) 17:1–8. 10.15420/ECR.2021.25 PMC8819604 35154392

[4] Bäck M Michel JB. From organic and inorganic phosphates to valvular and vascular calcifications. Cardiovasc Res. (2021) 117:2016–29. 10.1093/CVR/CVAB038 33576771 PMC8318101

[5] Rattazzi M Bertacco E Del Vecchio A Puato M Faggin E Pauletto P. Aortic valve calcification in chronic kidney disease. Nephrol Dial Transplant. (2013) 28:2968–76. 10.1093/NDT/GFT310 24097800

[6] Yamada S Giachelli CM. Vascular calcification in CKD-MBD: roles for phosphate, FGF23, and klotho. Bone. (2017) 100:87–93. 10.1016/J.BONE.2016.11.012 27847254 PMC5429216

[7] Hinton RB Yutzey KE. Heart valve structure and function in development and disease. Annu Rev Physiol. (2011) 73:29–46. 10.1146/ANNUREV-PHYSIOL-012110-142145 20809794 PMC4209403

[8] Leopold JA. Cellular mechanisms of aortic valve calcification. Circ Cardiovasc Interv. (2012) 5:605–14. 10.1161/CIRCINTERVENTIONS.112.971028 22896576 PMC3427002

[9] Miller JD Weiss RM Heistad DD. Calcific aortic valve stenosis: methods, models, and mechanisms. Circ Res. (2011). 108(11):1392–412. 10.1161/CIRCRESAHA.110.234138 21617136 PMC3150727

[10] Mohler ER. Mechanisms of aortic valve calcification. Am J Cardiol. (2004) 94:1396–402. 10.1016/J.AMJCARD.2004.08.013 15566910

[11] Osman L Yacoub MH Latif N Amrani M Chester AH. Role of human valve interstitial cells in valve calcification and their response to atorvastatin. Circulation. (2006) 114(1 Suppl):I-547–52. 10.1161/CIRCULATIONAHA.105.001115 16820635

[12] Goody PR Hosen MR Christmann D Niepmann ST Zietzer A Adam M Aortic valve stenosis: from basic mechanisms to novel therapeutic targets. Arterioscler Thromb Vasc Biol. (2020) 40:885–900. 10.1161/ATVBAHA.119.313067 32160774

[13] Greenberg HZE Zhao G Shah AM Zhang M. Role of oxidative stress in calcific aortic valve disease and its therapeutic implications. Cardiovasc Res. (2022) 118(6):1433–51. 10.1093/CVR/CVAB142 33881501 PMC9074995

[14] Li SJ Kao YH Chung CC Chen WY Li CW Chen YJ. Activated p300 acetyltransferase activity modulates aortic valvular calcification with osteogenic transdifferentiation and downregulation of klotho. Int J Cardiol. (2017) 232:271–9. 10.1016/j.ijcard.2017.01.005 28111052

[15] Wirrig EE Hinton RB Yutzey KE. Differential expression of cartilage and bone-related proteins in pediatric and adult diseased aortic valves. J Mol Cell Cardiol. (2011) 50:561–9. 10.1016/j.yjmcc.2010.12.005 21163264 PMC3035730

[16] Liberman M Bassi E Martinatti MK Lario FC Wosniak J Pomerantzeff PMA Oxidant generation predominates around calcifying foci and enhances progression of aortic valve calcification. Arterioscler Thromb Vasc Biol. (2008) 28:463–70. 10.1161/ATVBAHA.107.156745 18162610

[17] Clarke JA. An x-ray microscopic study of the blood supply to the valves of the human heart. Brit Hear J. (1965) 3:420–3 10.1136/hrt.27.3.420 PMC503325 14284360

[18] Weind KL Boughner DR Rigutto L Ellis CG. Oxygen diffusion and consumption of aortic valve cusps. Am J Physiol—Hear Circ Physiol. (2001) 281:2604–11. 10.1152/AJPHEART.2001.281.6.H2604/ASSET/IMAGES/LARGE/H41211185105.JPEG 11709429

[19] Charest A Pépin A Shetty R Côté C Voisine P Dagenais F Distribution of SPARC during neovascularisation of degenerative aortic stenosis. Heart. (2006) 92:1844–9. 10.1136/HRT.2005.086595 16709694 PMC1861285

[20] Katsi V Magkas N Antonopoulos A Trantalis G Toutouzas K Tousoulis D. Aortic valve: anatomy and structure and the role of vasculature in the degenerative process. Acta Cardiol. (2021) 76:335–48. 10.1080/00015385.2020.1746053 32602774

[21] Soini Y Salo T Satta J. Angiogenesis is involved in the pathogenesis of nonrheumatic aortic valve stenosis. Hum Pathol. (2003) 34:756–63. 10.1016/S0046-8177(03)00245-4 14506635

[22] Perrotta I Moraca FM Sciangula A Aquila S Mazzulla S. HIF-1*α* and VEGF: immunohistochemical profile and possible function in human aortic valve stenosis. Ultrastruct Pathol. (2015) 39:198–206. 10.3109/01913123.2014.991884 25569379

[23] Akahori H Tsujino T Naito Y Sawada H Sugahara M Fukui M Nuclear factor-*κ*B-hypoxia-inducible factor-2 pathway in aortic valve stenosis. J Heart Valve Dis. (2014) 23:558–66. https://scholars.uky.edu/en/publications/nuclear-factor-κb-hypoxia-inducible-factor-2-pathway-in-aortic-va (Accessed April 26, 2023). 25799704

[24] Syväranta S Helske S Laine M Lappalainen J Kupari M Mäyränpää MI Vascular endothelial growth factor-secreting mast cells and myofibroblasts: a novel self-perpetuating angiogenic pathway in aortic valve stenosis. Arterioscler Thromb Vasc Biol. (2010) 30:1220–7. 10.1161/ATVBAHA.109.198267 20299690

[25] Mazzone A Epistolato MC De Caterina R Storti S Vittorini S Sbrana S Neoangiogenesis, T-lymphocyte infiltration, and heat shock protein-60 are biological hallmarks of an immunomediated inflammatory process in end-stage calcified aortic valve stenosis. J Am Coll Cardiol. (2004) 43:1670–6. 10.1016/j.jacc.2003.12.041 15120829

[26] Rajamannan NM Nealis TB Subramaniam M Pandya S Stock SR Ignatiev CI Calcified rheumatic valve neoangiogenesis is associated with vascular endothelial growth factor expression and osteoblast-like bone formation. Circulation. (2005) 111:3296–301. 10.1161/CIRCULATIONAHA.104.473165 15956138 PMC3951870

[27] Chalajour F Treede H Ebrahimnejad A Lauke H Reichenspurner H Ergun S. Angiogenic activation of valvular endothelial cells in aortic valve stenosis. Exp Cell Res. (2004) 298:455–64. 10.1016/j.yexcr.2004.04.034 15265693

[28] Matilla L Martín-Núñez E Garaikoetxea M Navarro A Vico JA Arrieta V Characterization of the sex-specific pattern of angiogenesis and lymphangiogenesis in aortic stenosis. Front Cardiovasc Med. (2022) 9:971802. 10.3389/FCVM.2022.971802 36172587 PMC9510663

[29] Balogh E Tóth A Méhes G Trencsényi G Paragh G Jeney V. Hypoxia triggers osteochondrogenic differentiation of vascular smooth muscle cells in an HIF-1 (hypoxia-inducible factor 1)-dependent and reactive oxygen Species-dependent manner. Arterioscler Thromb Vasc Biol. (2019) 39:1088–99. 10.1161/ATVBAHA.119.312509 31070451

[30] Mokas S Larivière R Lamalice L Gobeil S Cornfield DN Agharazii M Hypoxia-inducible factor-1 plays a role in phosphate-induced vascular smooth muscle cell calcification. Kidney Int. (2016) 90:598–609. 10.1016/j.kint.2016.05.020 27470678

[31] Tóth A Csiki DM Nagy B Balogh E Lente G Ababneh H Daprodustat accelerates high phosphate-induced calcification through the activation of HIF-1 signaling. Front Pharmacol. (2022) 13:798053. 10.3389/FPHAR.2022.798053 35222025 PMC8867606

[32] Tani T Orimo H Shimizu A Tsuruoka S. Development of a novel chronic kidney disease mouse model to evaluate the progression of hyperphosphatemia and associated mineral bone disease. Sci Rep. (2017) 7(1):2233. 10.1038/s41598-017-02351-6 28533541 PMC5440375

[33] Fu B Wang J Wang L Wang Q Guo Z Xu M Integrated proteomic and metabolomic profile analyses of cardiac valves revealed molecular mechanisms and targets in calcific aortic valve disease. Front Cardiovasc Med. (2022) 9:944521. 10.3389/FCVM.2022.944521 36312243 PMC9606238

[34] Parra-Izquierdo I Castaños-Mollor I López J Gómez C San Román JA Sánchez Crespo M Lipopolysaccharide and interferon-*γ* team up to activate HIF-1*α* via STAT1 in normoxia and exhibit sex differences in human aortic valve interstitial cells. Biochim Biophys Acta Mol Basis Dis. (2019) 1865:2168–79. 10.1016/J.BBADIS.2019.04.014 31034990

[35] Wang J Zhang P Zhang J Ma Z Tian X Liu Y Atractylenolide-1 targets FLT3 to regulate PI3K/AKT/HIF1-*α* pathway to inhibit osteogenic differentiation of human valve interstitial cells. Front Pharmacol. (2022) 13:1455. 10.3389/FPHAR.2022.899775/BIBTEX PMC9097085 35571096

[36] Li XZ Xiong ZC Zhang SL Hao QY Gao M Wang JF Potential ferroptosis key genes in calcific aortic valve disease. Front Cardiovasc Med. (2022) 9:2104. 10.3389/FCVM.2022.916841/BIBTEX PMC9395208 36003913

[37] Nangaku M Eckardt KU. Hypoxia and the HIF system in kidney disease. J Mol Med. (2007) 85:1325–30. 10.1007/s00109-007-0278-y 18026918

[38] Gunaratnam L Bonventre JV. HIF In kidney disease and development. J Am Soc Nephrol. (2009) 20:1877–87. 10.1681/ASN.2008070804 19118148

[39] Norman JT Clark IM Garcia PL. Hypoxia promotes fibrogenesis in human renal fibroblasts. Kidney Int. (2000) 58:2351–66. 10.1046/J.1523-1755.2000.00419.X 11115069

[40] Manotham K Tanaka T Matsumoto M Ohse T Inagi R Miyata T Transdifferentiation of cultured tubular cells induced by hypoxia. Kidney Int. (2004) 65:871–80. 10.1111/J.1523-1755.2004.00461.X 14871406

[41] Querfeld U Mak RH Pries AR. Microvascular disease in chronic kidney disease: the base of the iceberg in cardiovascular comorbidity. Clin Sci (Lond). (2020) 134:1333–56. 10.1042/CS20200279 32542397 PMC7298155

[42] Babitt JL Lin HY. Mechanisms of anemia in CKD. J Am Soc Nephrol. (2012) 23:1631–4. 10.1681/ASN.2011111078 22935483 PMC3458456

[43] Salhiyyah K Sarathchandra P Latif N Yacoub MH Chester AH. Hypoxia-mediated regulation of the secretory properties of mitral valve interstitial cells. Am J Physiol Heart Circ Physiol. (2017) 313:H14–23. 10.1152/AJPHEART.00720.2016 28314761

[44] Swaminathan G Krishnamurthy VK Sridhar S Robson DC Ning Y Grande-Allen KJ. Hypoxia stimulates synthesis of neutrophil gelatinase-associated lipocalin in aortic valve disease. Front Cardiovasc Med. (2019) 6:156. 10.3389/FCVM.2019.00156 31737648 PMC6828964

[45] Kanno K Sakaue T Hamaguchi M Namiguchi K Nanba D Aono J Hypoxic culture maintains cell growth of the primary human valve interstitial cells with stemness. Int J Mol Sci. (2021) 22(19):10534. 10.3390/IJMS221910534 34638873 PMC8508607

[46] Wagegg M Gaber T Lohanatha FL Hahne M Strehl C Fangradt M Hypoxia promotes osteogenesis but suppresses adipogenesis of human mesenchymal stromal cells in a hypoxia-inducible factor-1 dependent manner. PLoS One. (2012) 7:e46483. 10.1371/JOURNAL.PONE.0046483 23029528 PMC3459928

[47] Ichijima T Matsuzaka K Tonogi M Yamane GY Inoue T. Osteogenic differences in cultured rat periosteal cells under hypoxic and normal conditions. Exp Ther Med. (2012) 3:165–70. 10.3892/ETM.2011.393/HTML 22969863 PMC3438792

[48] Park JH Park BH Kim HK Park TS Baek HS. Hypoxia decreases Runx2/Cbfa1 expression in human osteoblast-like cells. Mol Cell Endocrinol. (2002) 192:197–203. 10.1016/S0303-7207(02)00036-9 12088880

[49] Salim A Nacamuli RP Morgan EF Giaccia AJ Longaker MT. Transient changes in oxygen tension inhibit osteogenic differentiation and Runx2 expression in osteoblasts. J Biol Chem. (2004) 279:40007–16. 10.1074/jbc.M403715200 15263007

[50] Tamiya H Ikeda T Jeong JH Saito T Yano F Jung YK Analysis of the Runx2 promoter in osseous and non-osseous cells and identification of HIF2A as a potent transcription activator. Gene. (2008) 416:53–60. 10.1016/J.GENE.2008.03.003 18442887

[51] Tóth A Balogh E Jeney V. Regulation of vascular calcification by reactive oxygen Species. Antioxidants (Basel, Switzerland). (2020) 9:1–24. 10.3390/ANTIOX9100963 PMC7599480 33049989

[52] Branchetti E Sainger R Poggio P Grau JB Patterson-Fortin J Bavaria JE Antioxidant enzymes reduce DNA damage and early activation of valvular interstitial cells in aortic valve sclerosis. Arterioscler Thromb Vasc Biol. (2013) 33:66–74. 10.1161/ATVBAHA.112.300177 PMC4080420 23241403

[53] Smith KA Waypa GB Schumacker PT. Redox signaling during hypoxia in mammalian cells. Redox Biol. (2017) 13:228–34. 10.1016/J.REDOX.2017.05.020 28595160 PMC5460738

[54] Fernández-Agüera MC Gao L González-Rodríguez P Pintado CO Arias-Mayenco I García-Flores P Oxygen sensing by arterial chemoreceptors Depends on mitochondrial Complex I signaling. Cell Metab. (2015) 22:825–37. 10.1016/J.CMET.2015.09.004 26437605

[55] Waypa GB Smith KA Schumacker PT. O2 sensing, mitochondria and ROS signaling: the fog is lifting. Mol Aspects Med. (2016) 47–48:76–89. 10.1016/J.MAM.2016.01.002 PMC4750107 26776678

[56] Chandel NS Maltepe E Goldwasser E Mathieu CE Simon MC Schumacker PT. Mitochondrial reactive oxygen species trigger hypoxia-induced transcription. Proc Natl Acad Sci. (1998) 95:11715–20. 10.1073/PNAS.95.20.11715 9751731 PMC21706

[57] Rathore R Zheng YM Niu CF Liu QH Korde A Ho YS Hypoxia activates NADPH oxidase to increase [ROS]i and [Ca2+]i through the mitochondrial ROS-PKCɛ signaling axis in pulmonary artery smooth muscle cells. Free Radic Biol Med. (2008) 45:1223–31. 10.1016/J.FREERADBIOMED.2008.06.012 18638544 PMC2586914

[58] Jaakkola P Mole DR Tian YM Wilson MI Gielbert J Gaskell SJ Targeting of HIF-alpha to the von hippel-lindau ubiquitylation complex by O2-regulated prolyl hydroxylation. Science. (2001) 292:468–72. 10.1126/SCIENCE.1059796 11292861

[59] Nagy A Pethő D Gáll T Zavaczki E Nyitrai M Posta J Zinc inhibits HIF-prolyl hydroxylase inhibitor-aggravated VSMC calcification induced by high phosphate. Front Physiol. (2020) 10:1584. 10.3389/FPHYS.2019.01584 32009983 PMC6974455

[60] Eschbach JW Egrie JC Downing MR Browne JK Adamson JW. Correction of the Anemia of End-stage renal disease with recombinant human erythropoietin. N Engl J Med. (1987) 316:73–8. 10.1056/NEJM198701083160203 3537801

[61] Robles NR. The safety of erythropoiesis-stimulating agents for the treatment of Anemia resulting from chronic kidney disease. Clin Drug Investig. (2016) 36:421–31. 10.1007/s40261-016-0378-y 26894799

[62] McCullough PA Barnhart HX Inrig JK Reddan D Sapp S Patel UD Cardiovascular toxicity of epoetin-alfa in patients with chronic kidney disease. Am J Nephrol. (2013) 37:549–58. 10.1159/000351175 23735819

[63] Koulouridis I Alfayez M Trikalinos TA Balk EM Jaber BL. Dose of erythropoiesis-stimulating agents and adverse outcomes in CKD: a metaregression analysis. Am J Kidney Dis. (2013) 61:44–56. 10.1053/j.ajkd.2012.07.014 22921639 PMC3525813

[64] Palmer SC Navaneethan SD Craig JC Johnson DW Tonelli M Garg AX Meta-analysis: erythropoiesis-stimulating agents in patients with chronic kidney disease. Ann Intern Med. (2010) 153:23–33. 10.7326/0003-4819-153-1-201007060-00252 20439566

[65] Ishii T Tanaka T Nangaku M. Profile of daprodustat in the treatment of renal anemia due to chronic kidney disease. Ther Clin Risk Manag. (2021) 17:155–63. 10.2147/TCRM.S293879 33628028 PMC7898206

[66] Dhillon S. Daprodustat: first approval. Drugs. (2020) 80:1491–7. 10.1007/s40265-020-01384-y 32880805 PMC7471535

[67] Singh AK Carroll K McMurray JJV Solomon S Jha V Johansen KL Daprodustat for the treatment of Anemia in patients not undergoing dialysis. N Engl J Med. (2021) 385:2313–24. 10.1056/NEJMOA2113380 34739196

[68] Singh AK Carroll K Perkovic V Solomon S Jha V Johansen KL Daprodustat for the treatment of Anemia in patients undergoing dialysis. N Engl J Med. (2021) 385:2325–35. 10.1056/NEJMOA2113379 34739194

