# Supplementary material for: Hypoxia-inducible factor activation promotes osteogenic transition of valve interstitial cells and accelerates aortic valve calcification in a mice model of chronic kidney disease
Source: Front Cardiovasc Med. 2023 Jun 2;10:1168339. doi: 10.3389/fcvm.2023.1168339 (PMC10272757; doi:10.3389/fcvm.2023.1168339)
Supplement: Supplementary file 1 [file Datasheet1.docx]

**Supplementary information**

**Supplementary figure 1. The efficiency of HIF-1α and HIF-2α silencing**

(A-B) Confluent VICs were cultured in control (Ctrl) or osteogenic conditions (OM, 2.5 mmol/L excess Pi, 0.3 mmol/L excess Ca over Ctrl) in the presence of HIF-1α, HIF-2α or scrambled siRNA under normoxic condition. Protein expression of HIF-1α and HIF-2α in whole cell lysates (24h). (C-D) Confluent VICs were cultured in control (Ctrl) or osteogenic conditions in the presence of HIF-1α, HIF-2α or scrambled siRNA under hypoxic (1% O_2_) condition. Protein expression of HIF-1α and HIF-2α in whole cell lysates (24h). Membranes were re-probed for β-actin. Representative Western blots and relative expression of HIFs normalized to β-actin from 3 independent experiments. Data are expressed as mean ± SD. Ordinary one-way ANOVA followed by Tukey’s multiply comparison test was used to calculate p values. **p<0.01, ***p<0.005, ****p<0.001

**Supplementary figure 2. Kidney function parameters**

(A) Scheme of the experimental protocol. (B) Plasma urea, (C) plasma creatinine, (D) plasma phosphate levels in control (Ctrl) and CKD mice (n=4/group). Data are expressed as mean ± SD. t-test was used to calculate p values. *p<0.05 **p<0.01, ***p<0.005
