# Supplementary figures and images for: Hypoxia-inducible factor activation promotes osteogenic transition of valve interstitial cells and accelerates aortic valve calcification in a mice model of chronic kidney disease
Source: Front Cardiovasc Med. 2023 Jun 2;10:1168339. doi: 10.3389/fcvm.2023.1168339 (PMC10272757; doi:10.3389/fcvm.2023.1168339)

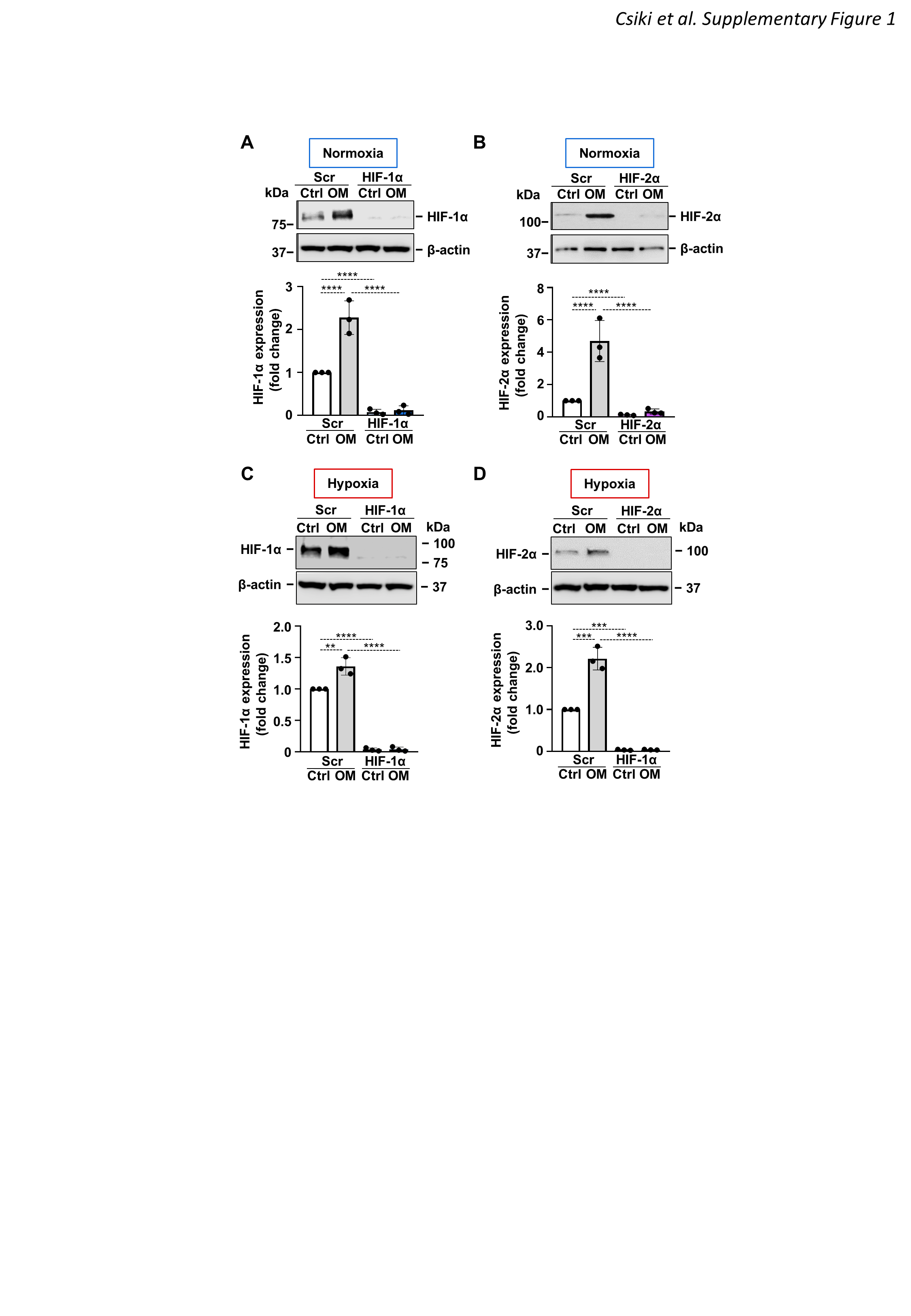

Supplement: Supplementary file 2 [file Image1.tif]

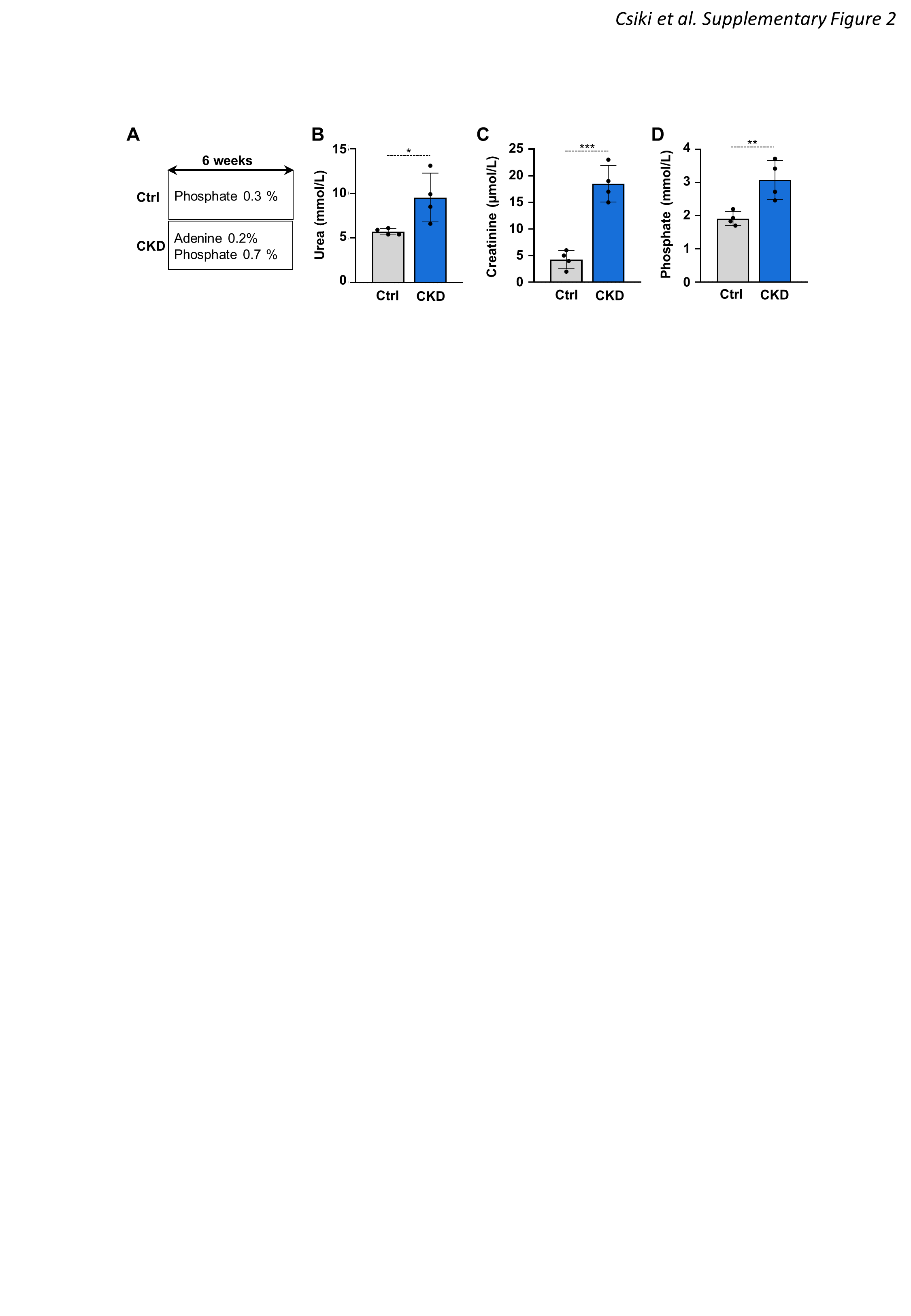

Supplement: Supplementary file 3 [file Image2.tif]
